# Supplementary figures and images for: HDAC6 inhibitor ACY-1083 shows lung epithelial protective features in COPD
Source: PLoS One. 2022 Oct 12;17(10):e0266310. doi: 10.1371/journal.pone.0266310 (PMC9555642; doi:10.1371/journal.pone.0266310)

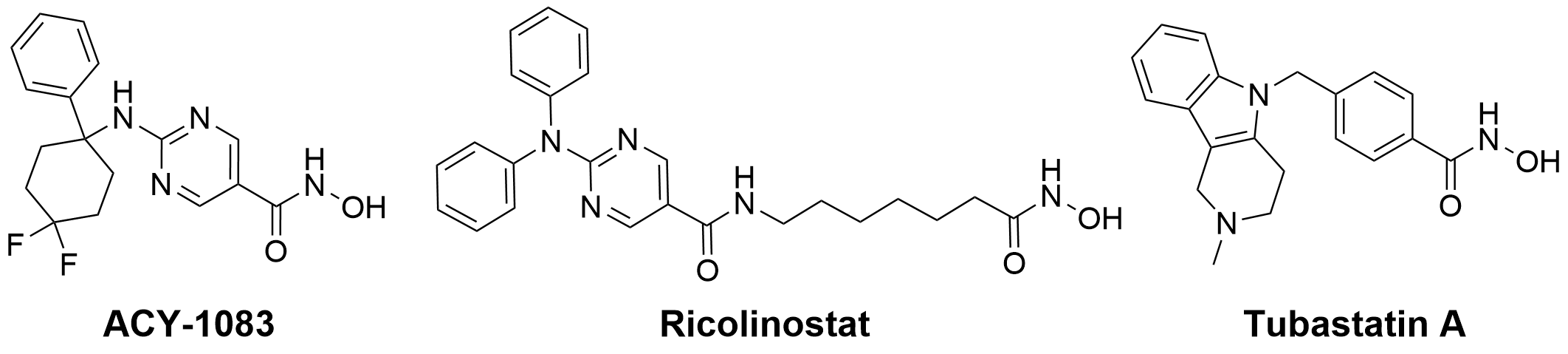

Supplement: S1 Fig — (TIF) [file pone.0266310.s001.tif]

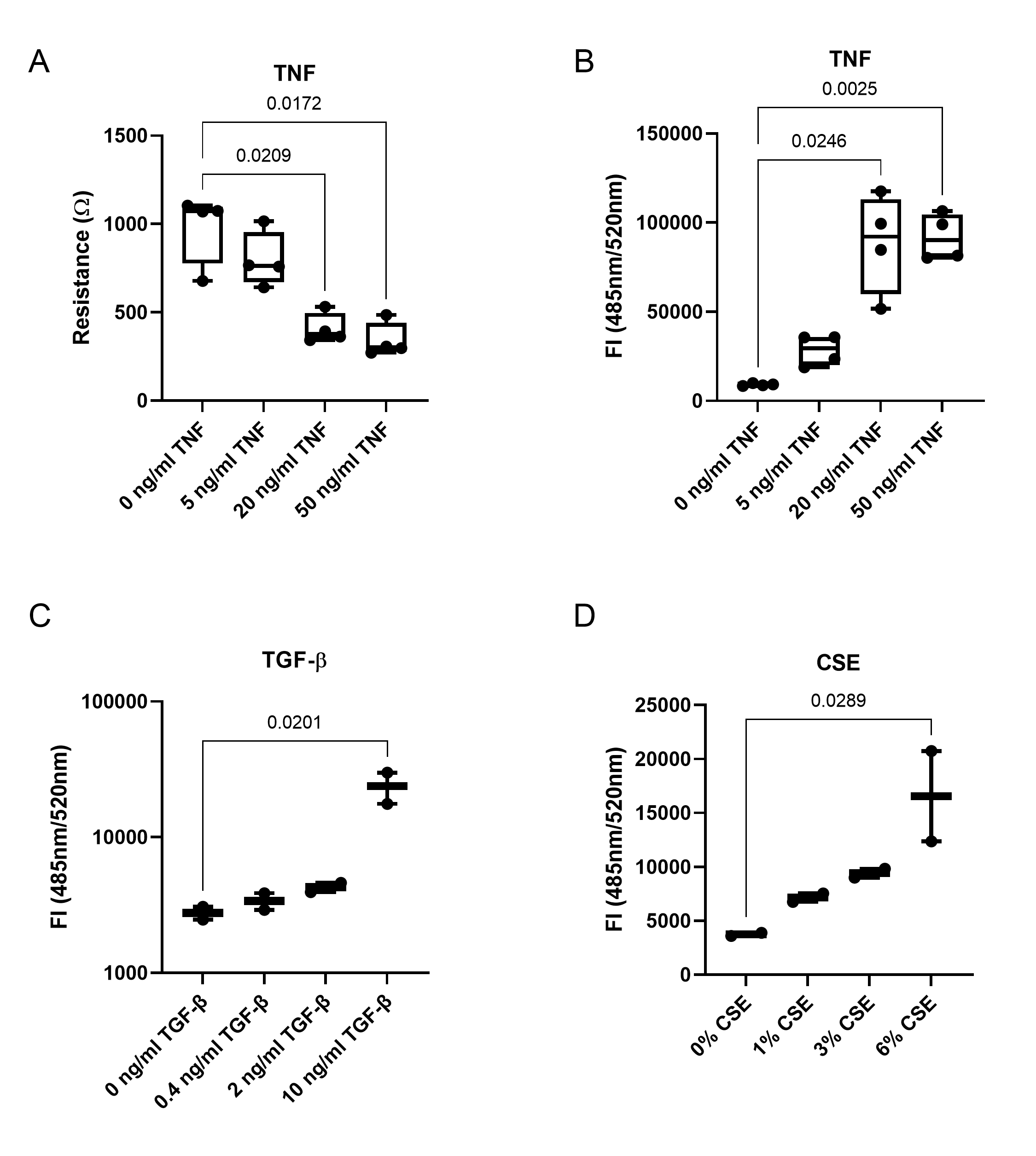

Supplement: S2 Fig — (A) Resistance measurements after 7 days of TNF-challenge (0–50 ng/ml). Box plot with dots representing the average of three replicates for each of 4 COPD donors and whiskers showing min and max. (B) FITC-Dextran measurement after 8 days of TNF challenge (0–50 ng/ml). Box plot with dots representing the average of three replicates for each of 4 COPD donors and whiskers showing min and max. (C) FITC-Dextran measurement after 2 days of TGF-β-challenge (0–10 ng/ml). Box plot with dots representing the average of three replicates for each of 2 COPD donors and whiskers showing min and max. (D) FITC-Dextran measurement after 48 hours CSE-challenge (0–6%). Box plot with dots representing the average of three replicates for each of 2 COPD donors and whiskers showing min and max. Statistical analysis was performed using one-way ANOVA with Dunnett´s multiple comparisons test. (TIF) [file pone.0266310.s002.tif]

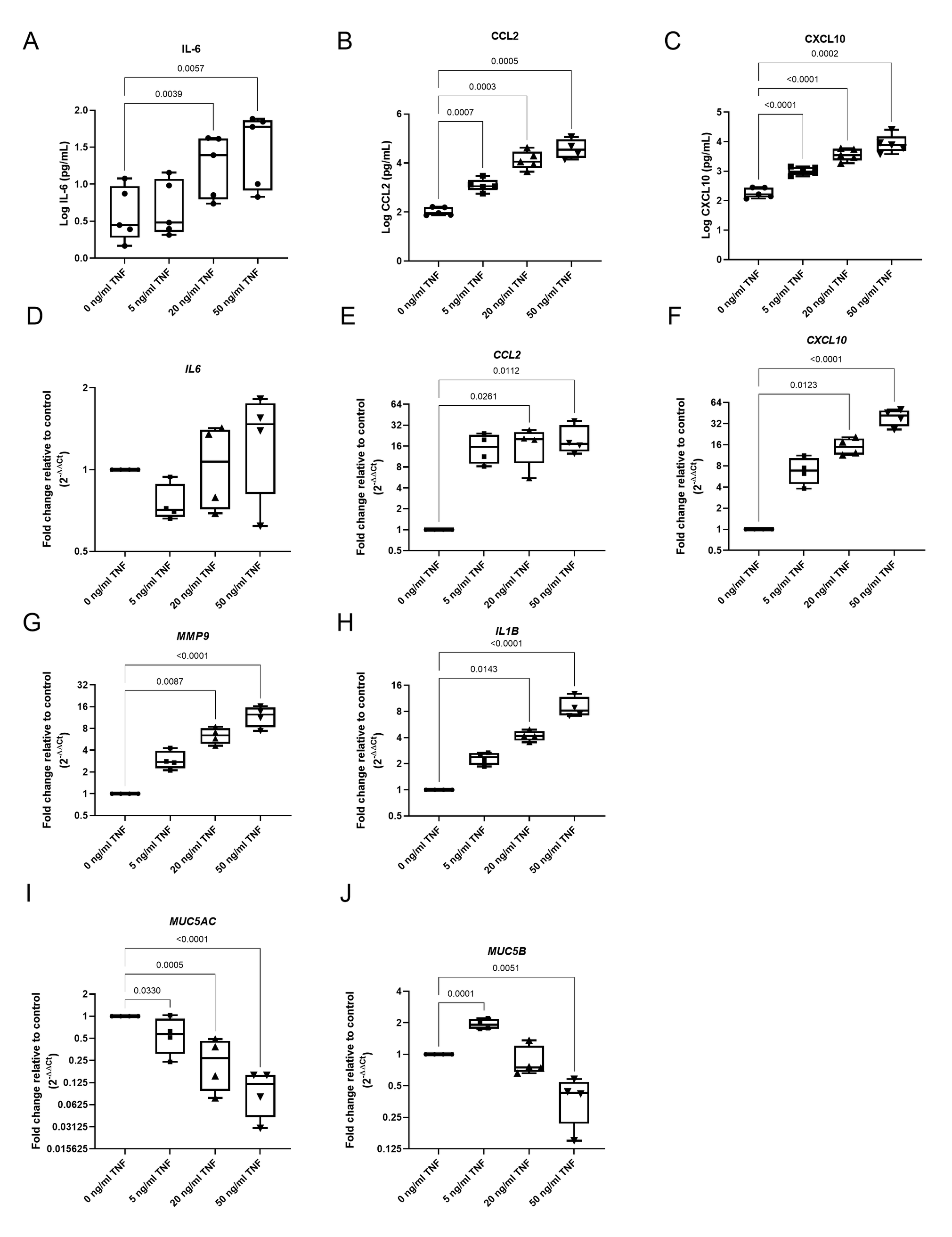

Supplement: S3 Fig — Protein concentrations in basolateral supernatants of (A) IL-6, (B) CCL2 and (C) CXCL10 in basolateral supernatants after 7 days of TNF-challenge measured by MSD. Relative mRNA levels of (D) IL6, (E) CCL2, (F) CXCL10, (G) MMP9, (H) IL1B, (I) MUC5AC and (J) MUC5B were assessed by RT-PCR from cells lysed after 8 days of TNF-challenge. Box plot with dots representing the average of three replicates for each of 4 COPD donors and whiskers showing min and max. Statistical analysis was performed using one-way ANOVA with Dunnett´s multiple comparisons test. (TIF) [file pone.0266310.s003.tif]

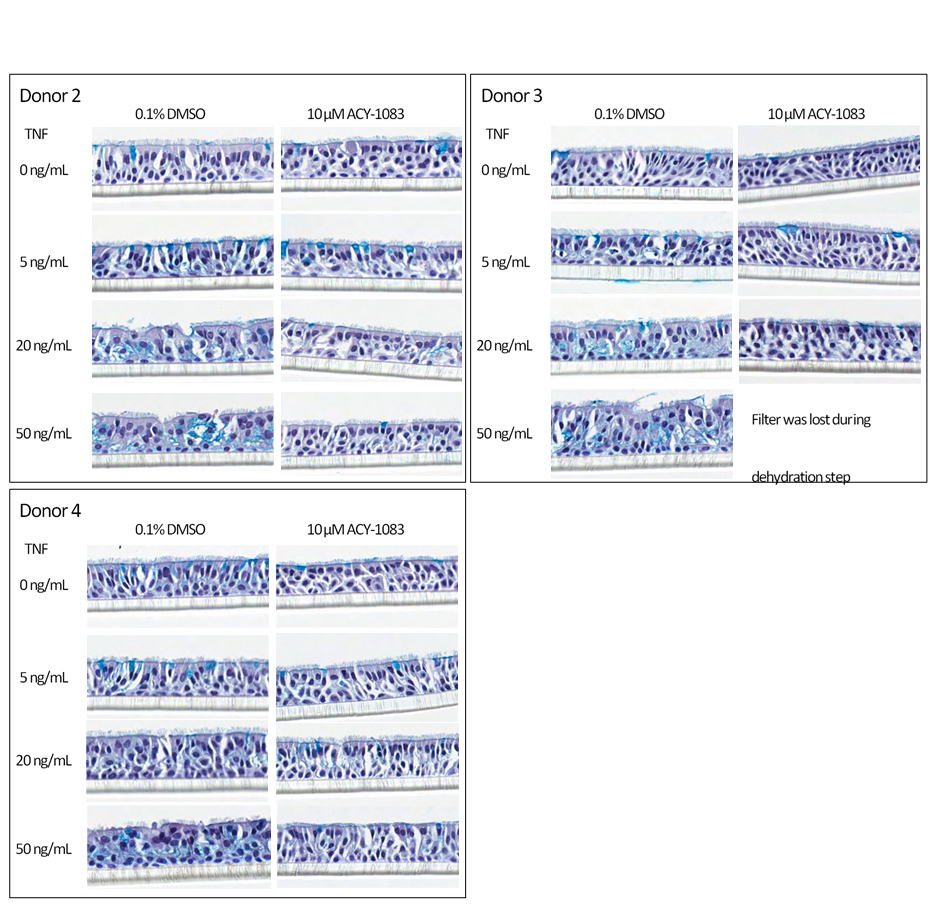

Supplement: S4 Fig — AB/PAS staining of sections of ALI cultures from 4 COPD donors challenged with different concentrations of TNF (0–50 ng/ml) with and without 10 μM ACY-1083. (TIF) [file pone.0266310.s004.tif]

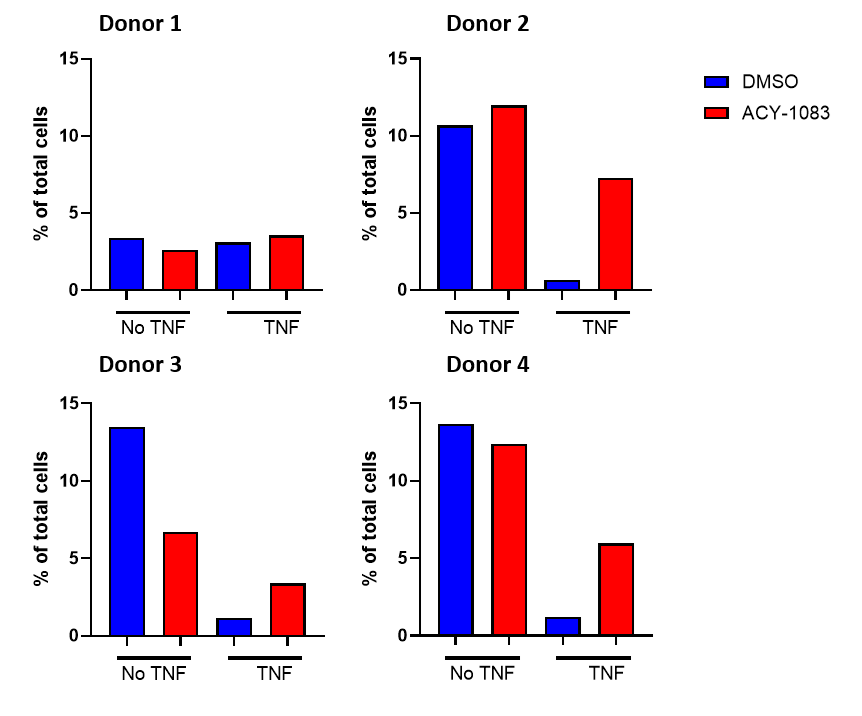

Supplement: S5 Fig — Quantification of goblet cell numbers in HBEC ALI sections stained with AB/PAS from 4 COPD donors. (TIF) [file pone.0266310.s005.tif]

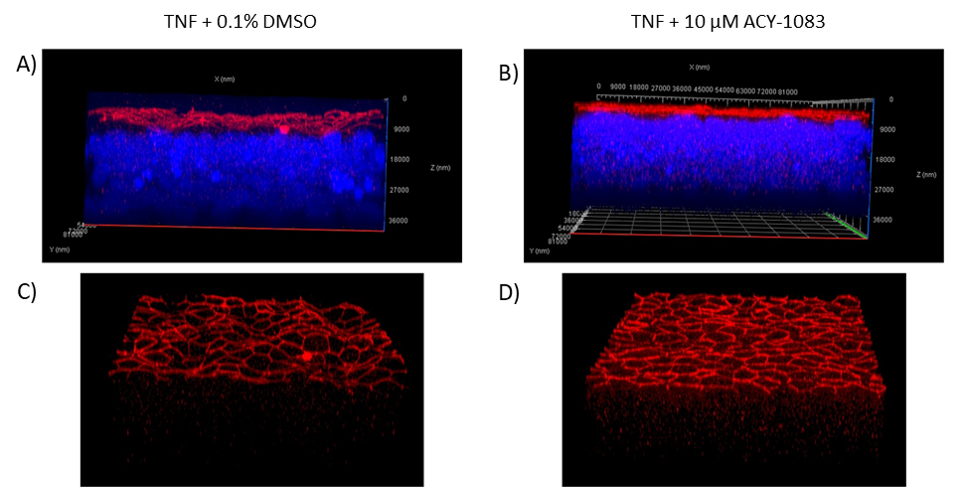

Supplement: S6 Fig — Occludin (in red) and nuclei (in blue) staining of HBEC ALI cultures from 1 COPD donor challenged with 20 ng/ml TNF. Images showing intersection of cell layer treated with A) vehicle or B) 10 μM ACY-1083. Occludin staining from the same sections as in A-B, C) showing vehicle and D) 10 μM ACY-1083. (TIF) [file pone.0266310.s006.tif]
