# Supplementary material for: HDAC6 inhibitor ACY-1083 shows lung epithelial protective features in COPD
Source: PLoS One. 2022 Oct 12;17(10):e0266310. doi: 10.1371/journal.pone.0266310 (PMC9555642; doi:10.1371/journal.pone.0266310)

Original blots for Figure 5 A-C

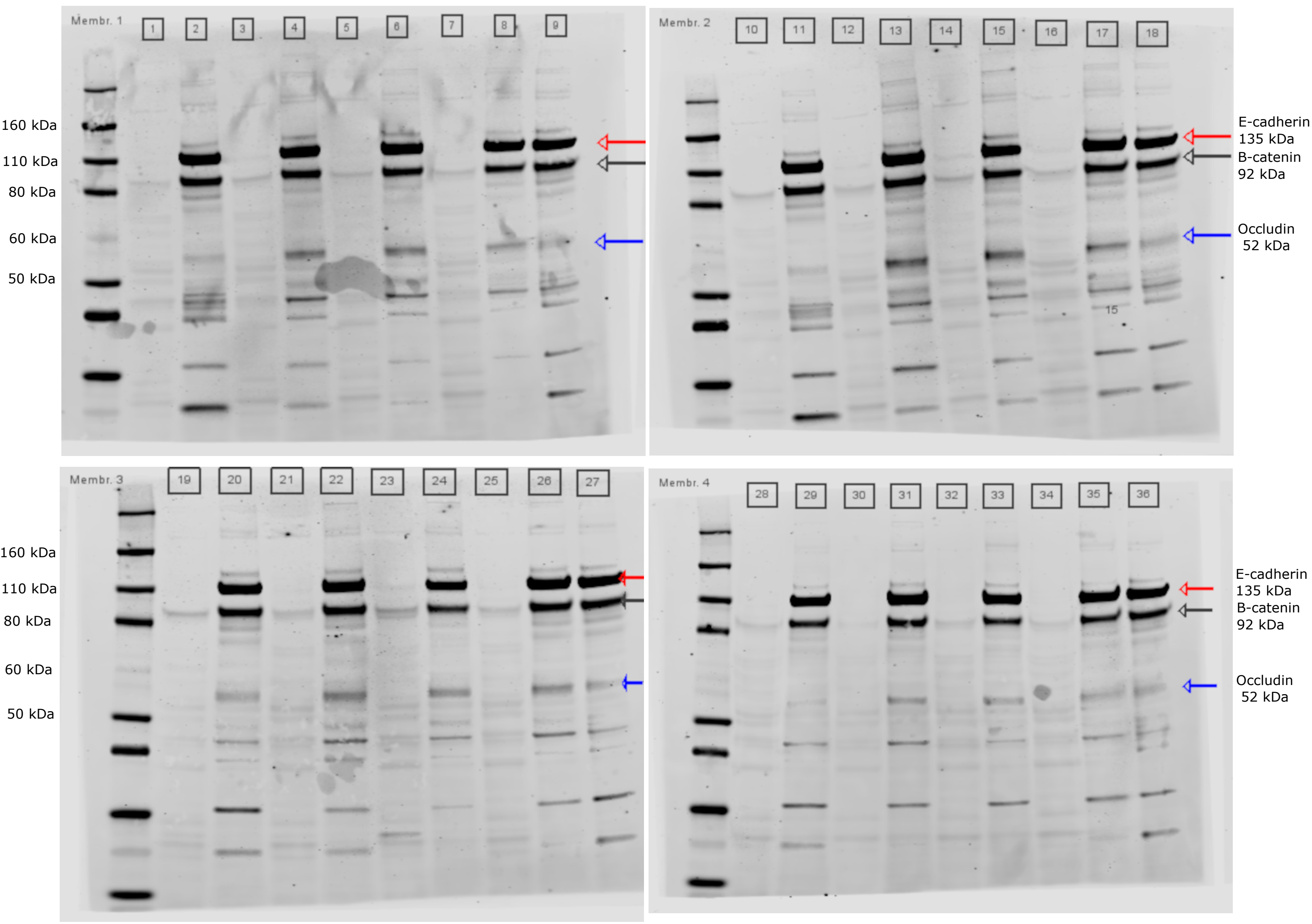

Membrane 1 (Donor 1)

1) Not used
2) DMSO 0.1%, no TNFa
3) Not used
4) ACY-1083 3.3 uM + TNFa
5) Not used
6) DMSO 0.1% + TNFa
7) Not used
8) ACY-1083 10 uM + TNFa
9) Intermembrane control

Membrane 2 (Donor 2)

10) Not used
11) DMSO 0.1%, no TNFa
12) Not used
13) ACY-1083 3.3 uM + TNFa
14) Not used
15) DMSO 0.1% + TNFa
16) Not used
17) ACY-1083 10 uM + TNFa
18) Intermembrane control

Membrane 3 (Donor 3)

19) Not used
20) DMSO 0.1%, no TNFa
21) Not used
22) ACY-1083 3.3 uM + TNFa
23) Not used
24) DMSO 0.1% + TNFa
25) Not used
26) ACY-1083 10 uM + TNFa
27) Intermembrane control

Membrane 4 (Donor 4)

28) Not used
29) DMSO 0.1%, no TNFa
30) Not used
31) ACY-1083 3.3 uM + TNFa
32) Not used
33) DMSO 0.1% + TNFa
34) Not used
35) ACY-1083 10 uM + TNFa
36) Intermembrane control

Novex™ Sharp Pre-stained Protein Standard

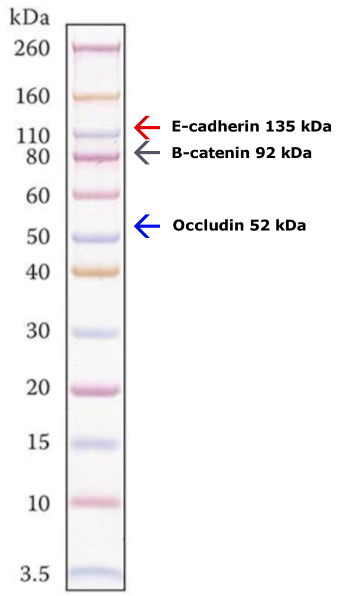

Supplement: S2 Raw images — (PDF) [file pone.0266310.s008.pdf]
