# Supplementary material for: HDAC6 inhibitor ACY-1083 shows lung epithelial protective features in COPD
Source: PLoS One. 2022 Oct 12;17(10):e0266310. doi: 10.1371/journal.pone.0266310 (PMC9555642; doi:10.1371/journal.pone.0266310)
Supplement: Attachment — Submitted filename: Response to reviewers.docx [file pone.0266310.s010.docx]

**ANSWERS TO REVIEWER’S COMMENTS**

PONE-D-22-07956R1

HDAC6 inhibitor ACY-1083 shows lung epithelial protective features in COPD

PLOS ONE

***Editors’ summary & main concerns:***

Thank you for submitting your manuscript to PLOS ONE. After careful consideration, we feel that it has merit but does not fully meet PLOS ONE’s publication criteria as it currently stands. Therefore, we invite you to submit a revised version of the manuscript that addresses the points raised during the review process.

The authors should contact Lonza to acquire additional information regarding the donors. This information is usually available upon request.

Thank you for this comment, we agree that it is very important information. We have contacted Lonza and we have the table of patient data that they have given us. Unfortunately there is no disease stage data. We have included the table further down in the response to Reviewer #2, first question.

The authors should remove the reference to cilia length as recommended by the Reviewer 2.

We have changed the manuscript accordingly, please see the response to Reviewer #2, second question for a detailed description of changes.

**Comments to the Author**

1. If the authors have adequately addressed your comments raised in a previous round of review and you feel that this manuscript is now acceptable for publication, you may indicate that here to bypass the “Comments to the Author” section, enter your conflict of interest statement in the “Confidential to Editor” section, and submit your "Accept" recommendation.

Reviewer #1: All comments have been addressed

Reviewer #2: (No Response)

2. Is the manuscript technically sound, and do the data support the conclusions?

The manuscript must describe a technically sound piece of scientific research with data that supports the conclusions. Experiments must have been conducted rigorously, with appropriate controls, replication, and sample sizes. The conclusions must be drawn appropriately based on the data presented.

Reviewer #1: Partly

Reviewer #2: Yes

3. Has the statistical analysis been performed appropriately and rigorously?

Reviewer #1: Yes

Reviewer #2: N/A

4. Have the authors made all data underlying the findings in their manuscript fully available?

The [PLOS Data policy](https://protect-de.mimecast.com/s/gEbGCnR2olClD1qQAcZIdrA?domain=plosone.org) requires authors to make all data underlying the findings described in their manuscript fully available without restriction, with rare exception (please refer to the Data Availability Statement in the manuscript PDF file). The data should be provided as part of the manuscript or its supporting information, or deposited to a public repository. For example, in addition to summary statistics, the data points behind means, medians and variance measures should be available. If there are restrictions on publicly sharing data—e.g. participant privacy or use of data from a third party—those must be specified.

Reviewer #1: Yes

Reviewer #2: Yes

5. Is the manuscript presented in an intelligible fashion and written in standard English?

PLOS ONE does not copyedit accepted manuscripts, so the language in submitted articles must be clear, correct, and unambiguous. Any typographical or grammatical errors should be corrected at revision, so please note any specific errors here.

Reviewer #1: Yes

Reviewer #2: Yes

6. Review Comments to the Author

Please use the space provided to explain your answers to the questions above. You may also include additional comments for the author, including concerns about dual publication, research ethics, or publication ethics. (Please upload your review as an attachment if it exceeds 20,000 characters)

Reviewer #1: Regarding to patient background, the number of donors, authors did not solve the problems fully, but described clearly (exploratory vs. main experiment) or provided best possible information. Also authors use very high concentration of compound for ALI, and from my experiences, the compound has problems in mucosal residency. This is an experimental drug, and all findings are for basic science rather than clinical trial mimic. Therefore, I am satisfied with all comments provided by authors. For future work, I wish authors conduct power calculation and use appropriate number of donors even for ALI work.

We thank Reviewer 1 for the careful assessment of the manuscript. We will in the future make sure we do power calculations.

Reviewer #2: The author have replied convincingly to the majority of my points. However, I still have a few remaining questions.

(1)Thank you for this important remark. Reviewer 1 has asked the same thing and we
write: The donors used, both the COPD and healthy bronchial epithelial cells are
originating from cells purchased from Lonza. We have very limited information about
these but we have included age, gender and smoking history for these donors on line
134.

I understand that the Lonza bought cells are difficult to get information on. However, the COPD stage is exceptionally important given the different responses you see amongst donors. I would still recommend that this is added.

We agree that this is important and the GOLD stage might as you are saying give us an explanation as to why the patients are responding differently to the treatments and stimulations. We have again contacted Lonza to ask for this information but they refer us to this table that I have also attached and say that they are not normally able to access information regarding GOLD stages. Had we been using our own patient cohorts or collaborations we would have been able to add this information but unfortunately this is not always the case for commercially available cells. We are sad to say that we have not been able to access the information asked for.

(2) We believe that with TNF as a stimulus, we don’t affect cilia length per se, but rather
affect the level of damage to the epithelium by inflammatory cytokines etc. It seems
that this is creating an uneven surface of the apical epithelium and that particles and
mucous get stuck and are not moving properly. We speculate that this is the reason
for CBF not to be affected by TNF, but possibly the synchronization of the beating is.
This is the reason why it is hard to show that ACY-1083 has a direct effect on cilia
length, but it should be mentioned that we lack the proper methods to measure this.

I would recommend removing any reference to cilia length in this case.

This is a good point. We have changed two sentences and thereby removed any specific mentioning of cilia length in the discussion.

“It is established that COPD patients have increased remodeling and production of mucins, shorter cilia and alterations in cilia-related genes” on line 580

and

“In line with these findings, a few papers have shown protective effects of HDAC6 inhibition on cilia destruction after challenges both *in vitro* and *in vivo*” on line 584
